# Root K Affinity Drivers and Photosynthetic Characteristics in Response to Low Potassium Stress in K High-Efficiency Vegetable Soybean

**DOI:** 10.3389/fpls.2021.732164

**Published:** 2021-10-21

**Authors:** Changkai Liu, Xue Wang, Bingjie Tu, Yansheng Li, Heng Chen, Qiuying Zhang, Xiaobing Liu

**Affiliations:** ^1^Key Laboratory of Mollisols Agroecology, Northeast Institute of Geography and Agroecology, Chinese Academy of Sciences, Harbin, China; ^2^Innovation Academy for Seed Design, Chinese Academy of Sciences, Harbin, China; ^3^University of the Chinese Academy of Sciences, Beijing, China

**Keywords:** potassium, potassium efficiency, bleeding sap, chlorophyll a/b ratio, vegetable soybean

## Abstract

Significant variations of potassium absorption and utilization exist in vegetable soybean. Pot and hydroponic experiments were carried out to examine the characteristics of root potassium (K) affinity-associated drivers and photosynthesis in vegetable soybean (edamame) [*Glycine max* (L.) Merr.] with different K efficiency. Two K high-efficiency vegetable soybean genotypes (Line 19 and Line 20) and two K low-efficiency genotypes (Line 7 and Line 36) were investigated in low K and normal K conditions. The root of K high-efficiency genotypes had a higher K^+^ affinity associated with a higher maximum K^+^ uptake rate (*I*max), but lower Michaelis constant for K^+^ absorption (*K*m) and lower compensation concentration for K^+^ uptake (*C*min). Seedlings of K high-efficiency genotypes also had higher root vigor [triphenyl tetrazolium chloride (TTC) reduction method] and greater absorbing activity (methylene blue method), especially in the low K condition. Furthermore, the root bleeding-sap rate of K high-efficiency genotypes in low K stress was 9.9–24.3% greater than that of normal K conditions, which was accompanied by a relatively higher K concentration of root bleeding-sap in contributing to K^+^ upward flux. The root of K high-efficiency vegetable soybean genotypes exhibited K^+^ high-affinity and driving advantages. Photosynthetic parameters of K high-efficiency vegetable soybean genotypes were less affected by low K stress. Low K stress decreased the net photosynthetic rate of K high-efficiency genotypes by 6.1–6.9%, while that of K low-efficiency genotypes decreased by 10.9–15.7%. The higher chlorophyll (Chl) a/b ratio with enhanced relative content of Chl a in response to low K stress might be an adapted mechanism for K high-efficiency genotypes to maintain photosynthetic capacity. Stronger root K affinity drivers associated with photosynthetic adaptability to low K stress are the key factors in determining the K high-efficiency of vegetable soybeans.

## Introduction

Potassium application benefits vegetable soybean yield and quality (Liu et al., 2017), while the direct absorption and utilization of available potassium by plants in cultivated soil are always essential (Singh and Reddy, 2017; Chen et al., 2020; Dev et al., 2021). Selecting and breeding potassium (K) efficient varieties of vegetable soybean is an important biological means in making full use of K resources (Rengel and Damon, 2008). Previous studies have shown that there are great differences in K efficiency among different genotypes. For instance, intraspecific variations in K efficiency have been reported in many crops including rice (Yang et al., 2003), wheat (Zhang et al., 1999; Damon and Rengel, 2007), sweet potato (Wang et al., 2015), tomato (Chen and Gabelman, 1995), and soybean (Sale and Campell, 1987; Wang C. et al., 2012; Liu et al., 2019b).

Differences in the K efficiency of crops can be understood from two main aspects, such as (1) the difference in K uptake efficiency and (2) the difference in K utilization efficiency (Rengel and Damon, 2008). The utilization efficiency of K refers to the ability of the crop to convert unit K into dry matter yield (Wang et al., 2018). K high-efficiency vegetable soybean genotypes are good at redistributing K and dry matters with higher harvest index (HI) and higher K harvest index (KHI) (Liu et al., 2019b). K uptake efficiency emphasizes the capacity of root K absorption (Tsialtas et al., 2017). The higher specific K uptake rate (total K content/total root length) in K high-efficiency vegetable soybean genotypes ensures the supply of K to the whole plant. Besides, K high-efficiency genotypes also have a strong ability to regulate their root architecture to adapt to low K conditions (Liu et al., 2019a).

Except for root morphology, K uptake kinetic parameters, root bleeding-sap, and root vigor are also demonstrated as effective parameters in evaluating K uptake efficiency (Teo et al., 1992; Hao et al., 2015; Cui et al., 2016; Zhang et al., 2017). Sufficient K can increase plant hydraulic conductance and transpiration (Pettigrew, 2008). The vigorous root can increase nutrient and water uptake, promoting the whole plant growth. For instance, the root bleeding-sap and the upward fluxes of K are higher in the cotton cultivars with high K efficiency (Yang, 2011). As substrates for photosynthesis, water and mineral elements absorbed by plant roots are transported upward by transpiration (Liu et al., 2016). Improved root characteristics may contribute to plant-water status, enhanced photosynthesis, biomass, and yield of soybean cultivars (Cui et al., 2016). In cotton, K efficient genotype 103 has more suitable K absorption kinetic parameters and more efficient photosynthate transport (Hao et al., 2015, 2016). Thus, the uptake power of the root system determines the supply of nutrients in the above-ground part of the plant.

Less photosynthetic assimilates and reduced assimilate transport out of the leaves to the developing fruit greatly contribute to the negative consequences that deficiencies of K have on yield and quality production (Pettigrew, 2008). Genotypic variations in photosynthetic decline caused by K deficiency have been reported. For instance, compared with the K-inefficient cotton cultivar, the K-efficient cultivar has a higher net photosynthetic rate (Pn) associated with higher biomass products (Wang N. et al., 2012).

Hence, understanding how plants take up and use K is of scientific and practical importance. Research into this mechanism has become increasingly urgent as a result of major ecological and agricultural issues. In our previous studies, K high-efficiency vegetable soybean genotypes were demonstrated with a strong K uptake and redistribution ability (Liu et al., 2019a,b). However, the root uptake dynamics and photosynthetic characteristics of K high-efficiency vegetable soybean are still not clear. Therefore, this study compared the differences of root activity, root bleeding-sap, the upward fluxes of K, K kinetics parameters, photosynthetic parameters, and chlorophyll content between K high-efficiency and K low-efficiency vegetable soybean genotypes. The data obtained revealed the mechanisms underlying K absorption and utilization of high-efficiency in vegetable soybean.

## Materials and Methods

### Plant Material

Based on previous K efficiency selection (Liu et al., 2019a), two K high-efficiency vegetable soybean genotypes (Line 19 and Line 20) and two K low-efficiency genotypes (Line 7 and Line 36) were used in this study. Compared with K low-efficiency genotypes, the K high-efficiency genotypes have higher K agronomic efficiency (KAE), recovery, internal utilization-efficiency rate (KIUE), and specific K uptake rate (Liu et al., 2019a,b). Greater reductions in K concentration of vegetative organs were found in K high-efficiency genotypes than K low-efficiency genotypes in low K conditions (Liu et al., 2019b). All genotypes were released by the Northeast Institute of Geography and Agroecology, Chinese Academy of Sciences, Harbin, China.

### Experimental Design

A pot experiment was conducted at the agronomy farm of Northeast Institute of Geography and Agroecology, Chinese Academy of Sciences, Harbin, China (45°732N, 126°612E; altitude 128 m a.s.l.) in 2017. The soil used was a typical Mollisol (Black soil) with the following properties: soil pH 6.6, organic matter 28.9 g kg^–1^, total N 2.3 g kg^–1^, total P 1.3 g kg^–1^, total K 18.9 g kg^–1^, available N 159 mg kg^–1^, available P 57.0 mg kg^–1^, and available K 85 mg kg^–1^ (insufficient for vegetable soybean yield and quality; Liu et al., 2017). The experiment was conducted in a completely random design with three replicates and five pots per replicate. About 3 seeds per pot were sown in May 2017 with regular manual pest and weed control. The pot size was 32 cm in diameter by 27 cm tall. A uniform fertilizer application at seeding included 70 kg ha^–1^ diammonium phosphate and 98 kg ha^–1^ urea. Treatments consisted of two K-fertilizer rates at seeding, K_2_SO_4_ 0 kg ha^–1^ (K0) and K_2_SO_4_ 120 kg ha^–1^ (K120). Plants were randomly harvested at fourth-node (V4), full bloom (R2), beginning seed (R5), and full seed (R6) stages (Fehr et al., 1971) for root bleeding-sap and K upward fluxes measurement.

A hydroponic experiment was used for K^+^ absorption kinetic parameters, root vigor, and root absorbing activity test. Seeds of the four selected genotypes were sterilized and germinated on moistened filter paper in a plant growth chamber at 60% humidity and 28°C, under a 16 h light, 8 h dark cycle for 3–4 days. After that, the seedlings were transferred into light-proof glass boxes (volume 15 cm^3^ × 20 cm^3^ × 20 cm^3^) with half-strength modified Hoagland nutrient solution, as described by Wang C. et al. (2012). There were six replications in each box. The cotyledons of all samples were excised to eliminate any additional supply of nutrients. When the plants grew to the first trifoliolate stage (V1), they were treated with half-strength Hoagland nutrient solution with different concentrations of K^+^ (0.5 and 3 mmol L^–1^). The K source was K_2_SO_4_. The initial pH value of the nutrient solution was 6, which was adjusted by 0.1 mol L^–1^ NaOH or 0.1 mol L^–1^ HCl. After transplanting, the samples were continuously cultured for 9 days. The nutrient solution was changed every 3 days and kept enough O_2_ by regular ventilation with an air pump. The pH value of the solution was adjusted to 6 when the nutrient solution was changed.

### Measurements

Collection of root bleeding-sap: root bleeding-sap collection was based on the method used by Cui et al. (2016) with slight improvements in the pot experiments. The root bleeding-sap was conducted from 9:30 a.m. to 1:30 p.m. at V4, R2, R5, and R6 stages. The plants were cut with branch scissors at cotyledons position, and the cross-section was cleaned with a small amount of absorbent cotton and then immediately connected to the root bleeding-sap collection device and the joint was sealed. The collected root bleeding-sap was temporarily stored in a refrigerator at 4°C for measurement. The formula was as follows: root bleeding-sap rate (g h^–1^ per plant) = bleeding-sap weight/4 h.

Where K upward fluxes = root bleeding-sap rate × K concentration, K concentration was determined by flame spectrophotometer (INESA FP6400A, Shanghai, China) (Liu et al., 2017; Nguyen et al., 2017).

K^+^ absorption kinetic parameters: A measurement of a depletion curve of a single plant. Five replicates per genotype were conducted. After K deficiency and starvation for 48 h, samples under the treatment of K3 at 9 days were immersed in 0.2 mmol L^–1^ CaSO_4_ solutions three times, dried, and put into a 200 ml absorption solution. The composition of the absorption solution was 0.05 mmol L^–1^ potassium chloride (KCl) + 0.2 mmol L^–1^ CaSO_4_. The absorption solution was placed in a dark conical flask, the culture temperature was (25 ± 2)°C, and the light intensity was 210–260 mol m^–2^ s^–1^. Parameter calculation including the maximum K^+^ uptake rate (*I*max), Michaelis constant for K^+^ absorption (Km), compensation concentration for K^+^ uptake (*C*min), and statistical analysis refers to the modified method of Epstein and Hagen (1952) and Claassen and Barber (1977). *C*min represents the concentration of K^+^ in the solution when the concentration remains constant. Ion consumption curve equation: Y = a + bX + cX^2^ (1), take the negative derivative of the equation: Y’ = b + 2cX (2). Hence, if X goes to 0, then Y’ = b = Km.

Root vigor, as determined by enzymatic reduction assay of triphenyl tetrazolium chloride (TTC) was conducted according to the modified protocol of Duncan and Widholm (2004).

Root absorption activity was determined by methylene blue adsorption (Zhang and Yan, 2003; Song and Wang, 2005). First, the root volume is converted to 1 ml = 1 g. The root was put into a solution with a known concentration of methylene blue for 1.5 min. Removed and placed the root into the second beaker, repeated twice. Colorimetric determination of methylene blue was conducted in the remaining solution in the three beakers at 660 nm using the UV-visible spectrophotometer (PERSEE T6, Beijing, China).


Totalabsorbingarea(m)2=[(C1-C1′)×V1]×1.1+[(C2-C2′)×V2]×1.1

Activelyabsorbingarea(m)2=[(C3-C3′)×V3]×1.1

$$\begin{array}{c} \mathrm{The}\mathrm{ratio}\mathrm{of}\mathrm{actively}\mathrm{absorbing}\mathrm{area}\mathrm{to}\mathrm{total}\mathrm{absorbing}\mathrm{area}\left(\%\right)\\ =100\times \mathrm{actively}\,\mathrm{absorbing}\,\mathrm{area}/\mathrm{total}\,\mathrm{absorbing}\,\mathrm{area} \end{array}$$

Theratioofthetotalabsorbingareatorootvolume(mcm2)-3=Total⁢absorbing⁢area/Root⁢volume

Where C, the original concentration of the solution (mg ml^–1^); C′, the concentration of the solution after leaching (mg ml^–1^); 1, 2, 3 is the beaker number; V, Volume of the solution; When 1 mg methylene blue forms a monolayer, it covers an area of 1.1 m^2^ (Zhang and Yan, 2003).

Photosynthetic properties of the youngest fully expanded main-stem leaf (the third leaf from the apex) were determined at 10:00–12:00 am at V4, R2, R4 (full pod), R5, and R6 stages with a Li-6800 (Li-COR, Lincoln, NE, United States) at 25°C, 60% relative humidity, 500 μmol mol^–1^ CO_2_ concentration, and 1,200 μmol m^–2^ s^–1^ quantum flux.

Determination of chlorophyll (Chl) content (Wang et al., 2021) was conducted using 0.2 g fresh leaves which were cut into pieces and put into a 50 ml centrifuge tube. Then, 20 ml of 80% ethanol solution was added and then soaked in a dark place for 8 h. Absorbance was determined at 665 and 649 nm, respectively, by the UV-visible spectrophotometer.


Chla=13.95A-6656.88A649Chlb=2.96A-6497.32A665Total⁢chlorophyll⁢concentration=Chl⁢a+Chl⁢b.

### Statistical Analyses

Differences among treatments and genotypes were examined by ANOVA using SPSS 17, and the means were separated by the LSD test at the 5% level. Linear regression analysis between root bleeding-sap rate per root length, K upward fluxes rate per root length, K concentration of root bleeding-sap, and plant K concentration were conducted after Pearson correlation analysis using a two-tailed test in SPSS 17. The figures were created using SigmaPlot 12.

## Results

### Comparison of K^+^ Absorption Kinetic Parameters Between Two K Efficiency Genotypes

Table 1 shows the K^+^ kinetics absorption parameters of the four vegetable soybean genotypes in a hydroponic experiment. The maximum K^+^ uptake rate (*I*max) in K high-efficiency genotypes was around 58.2–65.5 μmol g^–1^ min^–1^ FW, which was significantly higher than that of K low-efficiency genotypes around 42.4–54.1 μmol g^–1^ min^–1^ FW (*P* < 0.05). The compensation concentration for K^+^ uptake (*C*min) in K high-efficiency genotypes was 0.83–1.32 μmol L^–1^, which was lower than that of K low-efficiency genotypes (3.16–3.22 μmol L^–1^) (*P* <0.05). The Michaelis constant for K^+^ absorption (Km) is a parameter evaluating the affinity between root and K^+^. The greater the value, the smaller the affinity. Higher affinity was found in K high-efficiency vegetable soybean genotypes with a lower Km of 32.8–35.0 μmol L^–1^ than that of 48.6–49 μmol L^–1^ in K low-efficiency genotypes (*P* < 0.05).

**TABLE 1:** Comparison of K^+^ absorption kinetic parameters of root systems between two potassium (K) efficiency types under low K stress.

	**Line 19**	**Line 20**	**Line 7**	**Line 36**
Imax (μmol g^–1^ min^–1^ root FW^–1^)	65.0 a	58.2 b	42.4 d	54.1 c
Km (μmol L^–1^)	35.0 b	32.8 c	49.0 a	48.6 a
*C*min (μmol L^–1^)	1.32 b	0.83 c	3.16 a	3.22 a

*Different letters within the same row indicate statistical significance at the *P* < 0.05 level.*

### Root Bleeding-Sap and Upward Fluxes of K^+^ in the Pot Experiment

**FIGURE 1:**
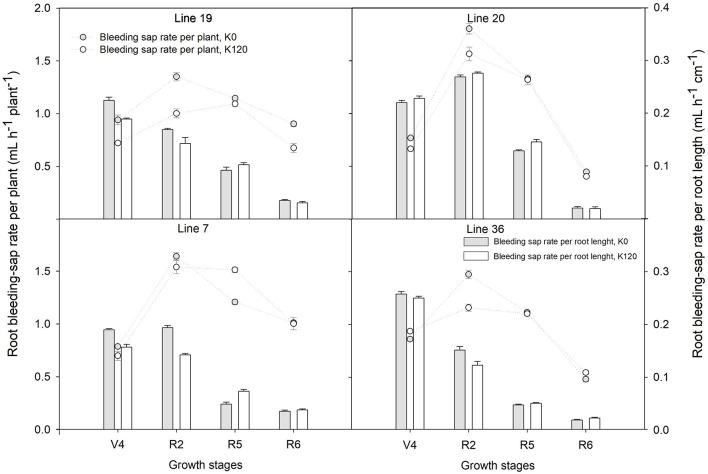
Root bleeding-sap of distinct potassium (K) efficiency genotypes under low K application in a pot experiment. Values are means of three replicates ± SE.

As shown in Figure 1, root bleeding-sap rate per plant showed a trend of increasing first and then decreasing from the fourth-node (V4) to full seed (R6) stage with the maximum at full bloom (R2) stage. The root bleeding-sap rate per plant of Line 20 was the highest among the four genotypes with 1.8 ml h^–1^ root^–1^ in K0 and 1.6 ml h^–1^ root^–1^ in K120, respectively. Compared with K120 treatment, K0 treatment increased the average of the root bleeding-sap rate per plant over the four stages by 9.9–24.3% in K high-efficiency genotypes, but by –2.2–5.1% in K low-efficiency genotypes. On the other hand, the root bleeding-sap rate per root length in four genotypes was found higher at the V4–R2 stage, dropped down at the R5–R6 stage. Interestingly, K high-efficiency genotypes had a higher root bleeding-sap rate per root length compared with K low-efficiency genotypes at the beginning seed stage (*P* < 0.05). At this time, root bleeding-sap rate per root length ranged 0.09–0.13 ml h^–1^ cm^–1^ in K0 and 0.10–0.15 ml h^–1^ cm^–1^ in K120 treatment, while in K low-efficiency genotypes kept around 0.05 ml h^–1^ cm^–1^ in K0 and 0.05–0.07 ml h^–1^ cm^–1^ in K120 treatment.

**FIGURE 2:**
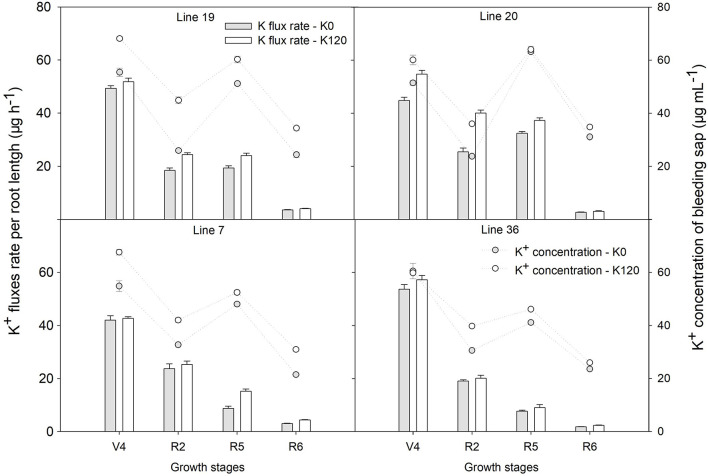
K upward fluxes and concentration in root bleeding sap between two K efficiency genotypes in a pot experiment. Values are means of three replicates ± SE.

Higher K upward fluxes were found in K120 treatment over the four genotypes, which was accompanied consistently by a higher concentration of K in the root bleeding-sap (Figure 2). The K flux rate per root length was highest at the fourth-node stage, with plant growth K flux rate per root length decreased. At the beginning seed stage, K high-efficiency genotypes had a higher K flux rate per root length and K concentration of root bleeding sap compared with K low-efficiency genotypes (*P* < 0.05). The K flux rate per root length of K high-efficiency genotypes was 19.4–32.4 μg h^–1^ cm^–1^ in K0 and 24.1–37.2 μg h^–1^ cm^–1^ in K120 treatment, that of 7.6–8.7 μg h^–1^ cm^–1^ in K0 and 9–15.2 μg h^–1^ cm^–1^ in K120 treatment in K low-efficiency genotypes. Meanwhile, K concentration of root bleeding-sap in K high-efficiency genotypes reached 51.1–63.2 μg ml^–1^ in K0 and 60.2–64 μg ml^–1^ in K120 treatment at the beginning seed stage, which was significantly higher than that of K low-efficiency genotypes with 41.1–48 μg ml^–1^ in K0 and 46.1–52.4 μg ml^–1^ in K120 treatment (*P* < 0.05).

Correlation analysis revealed that K upward fluxes rate per root length and K concentration of root bleeding-sap were positively correlated with K concentration per plant (*P* < 0.05) (Table 2).

**TABLE 2:** Correlation analysis of root bleeding-sap rate per root length, K upward fluxes rate per root length, K concentration of root bleeding-sap, and plant K concentration in vegetable soybean.

	**K upward fluxes rate per root length**	**K concentration of root bleeding-sap**	**Plant K concentration**
Root bleeding-sap rate per root length	0.884**	0.415*	0.707**
K upward fluxes rate per root length		0.745**	0.831**
K concentration of root bleeding-sap			0.598**

* *P* < 0.05, ** *P* < 0.01 for significance of correlations (Pearson). Plant K concentration data based on the study of Liu et al. (2019a).

### Root Vigor and Absorbing Activity

**FIGURE 3:**
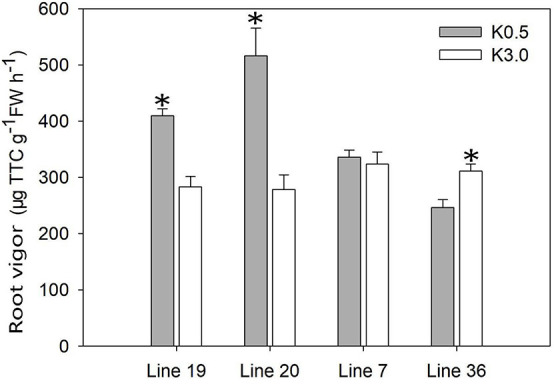
Effect of low K treatment on root vigor of vegetable soybean with different K efficiency in a hydroponic experiment. Values are means of three replicates ± SE. ^∗^means significant differences at *P* < 0.05 level.

The root vigor was tested in a hydroponic experiment using the measurement of respiratory activity with triphenyl tetrazolium chloride (TTC) (Figure 3). At the seedling stage (9 days after treatment), the root vigor of K high-efficiency genotypes was induced by low K stress. Low K stress (K0.5) increased the root vigor of K high-efficiency genotypes by 46–85% compared with normal K treatment (K3) (*P* < 0.05). Although no consistent trend was observed in K low-efficiency genotypes, low K stress decreased root vigor by 21% in Line 36 (*P* < 0.05). The highest root vigor of 516 μg g^–1^ FW h^–1^ was found in Line 20 under low K conditions.

The root absorbing activity parameters are shown in Table 3. Higher total absorbing area and actively absorbing area were found in K high-efficiency genotypes. Meanwhile, in K high-efficiency genotypes, low K stress increased the actively absorbing area by 21.4–30.6% and the total absorbing area by 9.6–19% (*P* < 0.05). In contrast, an opposite trend was found in K low-efficiency genotypes. Under low K stress, the actively absorbing area decreased by 6.1–10.3% and the total absorbing area decreased by 6.6–15.7% (*P* > 0.05). Furthermore, K high-efficiency genotypes also had a higher ratio of total absorbing area to root volume, especially in Line 19 with 62.3 m^2^ cm^–3^ (K0.5) and 60.2 m^2^ cm^–3^ (K3). Low K stress increased the ratio of actively absorbing area to total absorbing area by 9.3–9.4% in K high-efficiency genotype (*P* < 0.05), but by 0.6–5% in K low-efficiency genotypes (*P* > 0.05).

**TABLE 3:** Root adsorption activity of vegetable soybean genotypes with different K efficiency.

		**Total absorbing area (m^2^ plant^–1^)**	**Actively absorbing area (m^2^ plant^–1^)**	**Ratio of actively absorbing area to total absorbing area (%)**	**Ratio of total absorbing area to root volume (m^2^ cm^–3^)**
Line 19	K0.5	0.103a	0.051a	49.5a	62.3a
	K3.0	0.094b	0.042*bc*	45.3*bc*	60.2a
Line 20	K0.5	0.094b	0.047*ab*	49.9a	52.9b
	K3.0	0.079c	0.036cd	45.6bc	52.5b
Line 7	K0.5	0.070d	0.035de	50.1a	52.1b
	K3.0	0.083c	0.039cd	47.7ab	44.2c
Line 36	K0.5	0.071d	0.031e	43.9c	40.6cd
	K3.0	0.076cd	0.033de	43.6c	38.8d

*Different letters within the same column indicate statistical significance at the *P* < 0.05 level.*

### Comparison of Photosynthetic Parameters Between Two K Efficiency Genotypes

**FIGURE 4:**
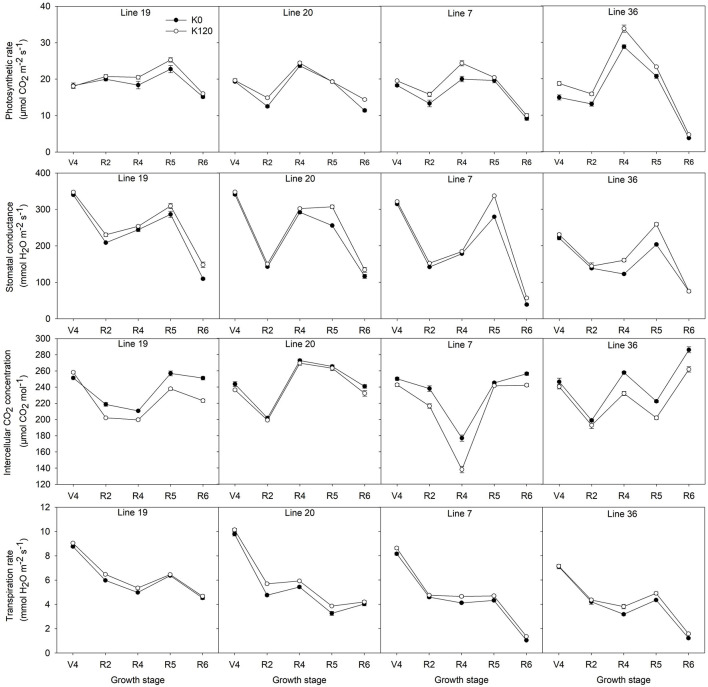
Changes of the photosynthetic parameters of vegetable soybean genotypes with different K efficiency in a pot experiment. Values are means of three replicates ± SE.

A pot experiment was conducted to determine photosynthetic parameters of two K efficiency vegetable soybean genotypes affected by K deficiency (K0) are shown in Figure 4. K deficiency decreased the photosynthetic rate (Pn) of the four vegetable soybean genotypes. During the whole growth stages, the Pn of K high-efficiency genotypes decreased by 6.1–6.9%, while that of low-efficiency genotypes decreased by 10.9–15.7%. From full bloom to full seed stage, the Pn increased first and then decreased. The maximum value occurred at the full pod (R4) stage of the K low-efficiency genotype Line 36, which reached 28.8 μmol CO_2_ m^–2^ s^–1^ and 33.8 μmol CO_2_ m^–2^ s^–1^ under K0 and K120 treatments, respectively. Meanwhile, from full pod to full seed stage, the dramatic decline of the Pn was found in K low-efficiency genotypes. The Pn of Line 36 decreased by 87.2% in K0 and 86.2% in K120 treatment (*P* < 0.05), and that of Line 7 decreased by 54.3% in K0 and 59% in K120 treatment. Whereas the decline of the Pn in K high-efficiency genotypes was around 33.5–51.8% in K0 and 36.7–41.1% in K120 treatment, which was not as much as that of K low-efficiency genotypes.

Potassium deficiency also decreased the stomatal conductance (Gs) in all genotypes, but K high-efficiency genotypes were less affected. The decrease of Gs by K deficiency was 7.5–7.8% in K high-efficiency genotypes, but 9.4–12.5% in K low-efficiency genotypes. Low K treatment had the most obvious effect on Gs at the beginning seed stage.

As opposed to Pn and Gs, K deficiency increased the intercellular CO_2_ concentration (Ci), especially in K low-efficiency genotypes. The increase of Ci by K deficiency was 7.3–7.9% in K low-efficiency genotypes, but 2–6% in K high-efficiency genotypes.

Potassium deficiency decreased the transpiration rate (Tr) of all genotypes. Across the five stages, Tr of K high-efficiency genotypes was around 8.8–9.8 mmol H_2_O m^–2^ s^–1^ in K0 and 9–10.1 mmol H_2_O m^–2^ s^–1^ in K120 treatment, which was higher than that of 7.1–8.2 mmol H_2_O m^–2^ s^–1^ in K0 and 7.1–8.6 mmol H_2_O m^–2^ s^–1^ in K120 treatment in K low-efficiency genotypes. At the full seed stage, higher Tr was found in K high-efficiency genotypes with 4–4.5 mmol H_2_O m^–2^ s^–1^ in K0 and 4.2–4.7 mmol H_2_O m^–2^ s^–1^ in K120 treatment, but only 1–1.2 mmol H_2_O m^–2^ s^–1^ and 1.4 –1.6 mmol H_2_O m^–2^ s^–1^ in K low-efficiency genotypes.

### Chlorophyll

Pot experiment indicated that K deficiency significantly decreased leaf chlorophyll content of the four vegetable soybean genotypes (Table 4). The average total Chl content in the four growth stages of K high-efficiency genotypes was 2.29–2.53 mg g^–1^ in K0 and 2.89–2.91 mg g^–1^ in K120 treatment, which was lower than that of K low-efficiency genotypes with 2.57–3.2 mg g^–1^ in K0 and 3.08–3.5 mg g^–1^ in K120 treatment. However, K deficiency increased the ratio of Chl a to Chl b, especially in K high-efficiency genotypes. The ratio of Chl a to Chl b was increased by 36.2–38.1% in K high-efficiency genotypes (*P* < 0.05), but only by 6–9.8% in K low-efficiency genotypes.

**TABLE 4:** Changes of chlorophyll a, chlorophyll b, and total chlorophyll concentrations (mg g^–1^) of vegetable soybean with different K efficiency in a pot experiment.

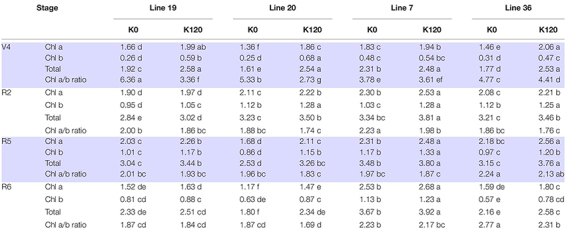

*Different letters within the same row indicate statistical significance at the *P* < 0.05 level.*

## Discussion

Potassium high-efficiency vegetable soybean genotypes are more efficient in root architecture adjustment associated with higher specific root K uptake rate (total K accumulation/total root length) to adapt to low K conditions, which ensures an adequate supply of K (Liu et al., 2019a). Based on these, this study investigated the root bleeding-sap rate and K upward fluxes rate of K high and low-efficiency vegetable soybean genotypes, which are important indicators assessing root pressure, root activity, and K uptake abilities (Doussan et al., 2006; Wang P. et al., 2020). The results indicated that K high-efficiency vegetable soybean genotypes against low K condition by increasing root bleeding-sap rate per plant and maintaining higher root bleeding-sap rate per root length at the beginning seed stage. The root bleeding-sap rate of K high-efficiency genotypes in low K stress was 9.9–24.3% greater than that of normal K conditions, which was accompanied by the relatively higher K concentration of root bleeding-sap in contributing to K^+^ upward flux. In K high-efficiency cotton cultivars, higher root bleeding-sap and K upward fluxes could also be induced by low K stress (Yang, 2011). Suitable grafting would help watermelon seedlings accumulate more K by increasing root bleeding-sap volume and the total K in the root bleeding-sap (Huang et al., 2013). Due to the rate of root bleeding-sap is closely related to plant nutrient supply, water transport, and even photosynthesis (Guan et al., 2014; Jia et al., 2018; He et al., 2019), increased rate of root bleeding-sap under K deficiency might be an important regulatory mechanism for vegetable soybean efficient uptake K. Besides, beginning seed stage is a most important period for seed establishment, the higher root bleeding-sap rate per root length in K high-efficiency vegetable soybean genotypes accompanied by higher K upward fluxes rate per root length and higher K concentration of root bleeding-sap is positively correlated with plant K concentration (*P* < 0.05). This is another evidence demonstrating that the root of K high-efficiency genotypes has a stronger affinity for K^+^.

Potassium kinetic parameters and root activities are also important factors assessing K efficiency when plants suffering low K stress (Silberbush and Barber, 1983; Cui et al., 2016; White et al., 2018). The present study revealed a lower Michaelis constant (*K*m) and compensation concentration for K^+^ uptake (*C*min), and a higher maximum K^+^ uptake rate (*I*max) from K high-efficiency genotypes, compared with K low-efficiency genotypes. Lower *K*m and *C*min indicate higher affinity between the roots and K^+^ and stronger ability to use the low-concentration K^+^, while higher *I*max ensures a faster K uptake rate (Glass, 1980; Teo et al., 1992). The absorption kinetic parameters of ion uptake are useful indexes of the level of adaptation of the genotype to the nutrient condition in the soil (Crowley, 1975; Daniel et al., 2020). The study of Hao et al. (2015) also recognized that K kinetic parameters could be used to test crop low-K adaptability. Therefore, it was reasonable to say that K high-efficiency genotypes had better K adaptability than K low-efficiency genotypes. On the other hand, root activity is another important heritable trait in evaluating root absorption ability, which also influences nutrients acquisition and initial canopy cover and, thereby, crop yields (Liu et al., 2015; Cui et al., 2016; White et al., 2018). In the present study, both root vigor and absorbing activity (including actively absorbing area, total absorbing area, and the ratio of actively absorbing area to total absorbing area) were consistently enhanced by low K stress in K high-efficiency genotypes, while those of K low-efficiency genotypes are inhibited. This kind of difference can be regulated by plant phytohormones (Yang, 2011), which were controlled by specific genes and pathways (Liu et al., 2020). Therefore, K high-efficiency genotypes are more adapted to low K stress through regulating root K affinity drivers, which is beneficial for the upward flux of nutrients (Liu et al., 2015).

Photosynthetic parameters affected by K levels are direct references to characterize the photosynthetic capacity of crops (Wang Y. et al., 2020). Hence, it is important to examine the photosynthetic characteristics of vegetable soybean with different K efficiency types. In the present study, photosynthetic parameters are less affected by low K in K high-efficiency genotypes. The *Pn* of K high-efficiency genotypes decreased by 6.1–6.9% and K low-efficiency genotypes by 10.9–15.7%. Many investigations also indicate that crops with high K efficiency should be more efficient in photosynthesis or that photosynthetic capacity is less affected by low K stress (Wang et al., 2014). For instance, under K deficiency, *Pn* of K-efficient cotton cultivar Liaomian 18 is 19.4% higher than that of K-inefficient cultivar NuCOTN99^B^. Besides, photosynthetic parameters of Liaomian 18 were less affected by low K stress (Wang N. et al., 2012). However, the present study did not find absolute superiority of *Pn* in K high-efficiency genotypes, but they have a higher HI and KHI [the relevant results have been published by Liu et al. (2019b)]. A high HI is fundamental to efficient utilization of all resources taken up by the plant, and the photosynthate transport and distribution rather than photosynthesis rate are critical for low K adaptation in K high-efficiency genotypes (Rengel and Damon, 2008; Hao et al., 2016).

Consistent with photosynthetic parameters, Chl content also reflects the photosynthetic activity in leaves (Szafrańska et al., 2017; Choudhury et al., 2019). In the present study, K high-efficiency vegetable soybean genotypes exhibit lower total Chl content but greater Chl a/b ratio when suffering low K stress. The effect of photosynthetic photon flux density on the leaf Chl a/b ratio is one of the most characteristic differences between sun and shade leaves (Abtahi et al., 2019). Typically, total Chl content per unit leaf area is lower and the Chl a/b ratio is greater in sun compared with shade soybean leaves (Anderson, 1986; Fritschi and Ray, 2007). The higher Chl a/b ratio with enhanced relative content of Chl a in response to low K stress might be an adapted mechanism for K high-efficiency genotypes to maintain photosynthetic capacity because Chl a is the main pigment in leaves that absorbs light energy, which ensures light absorption as much as possible (Abtahi et al., 2019). Similar results were also revealed in the research of Wang et al. (2008). Besides, low K stress decreased leaf K^+^ concentration of vegetable soybean, but K high-efficiency genotypes were less affected (Liu et al., 2019a). This could be an important internal factor affecting the chlorophyll regulation ability of K high-efficiency vegetable soybean. The present study recognized that the high efficiency of photosynthesis, including more adaptable photosynthetic parameters and Chl proportion, was essential for K utilization efficiency in K high-efficiency vegetable soybean genotypes.

It is root uptake power that drives up nutrients transport, while photosynthetic capacity and assimilate redistribution capacity are the key to crop yield (He et al., 2019). In the present study, K high-efficiency vegetable soybean was found to have obvious advantages in root K affinity drivers, which ensured the upward supply of K and other nutrients. Thus, the photosynthetic system of K high-efficiency genotypes was less susceptible to low K conditions and has a stronger regulation ability, ensuring the efficiency of K utilization.

## Conclusion

**FIGURE 5:**
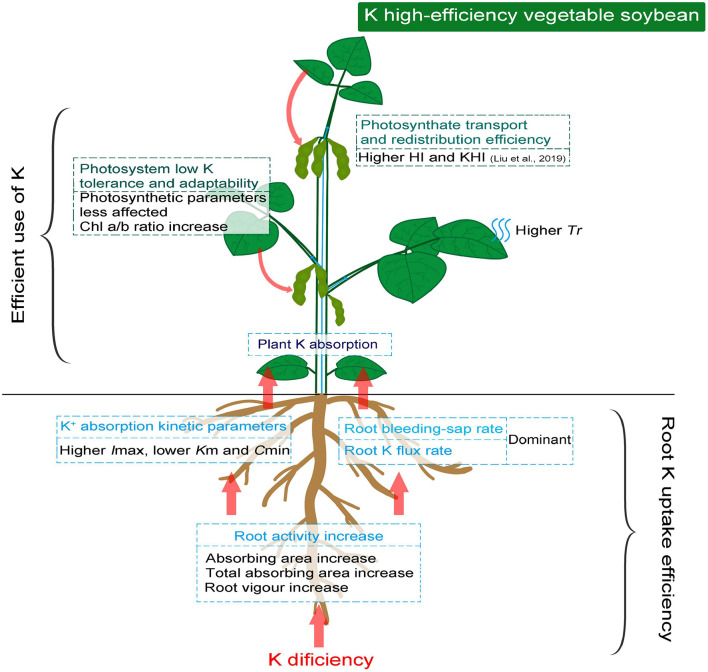
Schematic diagram demonstrating efficient K absorption and photosynthetic assimilation in K high-efficiency vegetable soybean under low K condition.

The higher affinity of root to K^+^ associated with root activity under low K stress is essential to promote root K absorption. The strong drivers, represented with higher root bleeding-sap rate and Tr induced by low K stress, ensure the upward flux of K^+^ and other essential nutrients as much as possible. The photosynthetic system of K high-efficiency vegetable soybean genotypes is less affected and reasonably regulated by low K stress to maintain photosynthates. Therefore, the ability to redistribute photosynthetic products seemed more important than photosynthetic capacity in K high-efficiency genotypes (Figure 5). Overall, crop K high-efficiency should be holistic, and the factors involved are not isolated. Stronger root K affinity drivers associated with photosynthetic adaptability to low K stress were the key factors in determining the K high-efficiency of vegetable soybeans.

## Data Availability Statement

The raw data supporting the conclusions of this article will be made available by the authors, without undue reservation.

## Author Contributions

XL and QZ designed the experiments, supervised the study, and revised the manuscript. CL and XW performed the research and wrote the manuscript. HC, BT, and YL helped in planting and data analysis. All authors contributed to the article and approved the submitted version.

## Conflict of Interest

The authors declare that the research was conducted in the absence of any commercial or financial relationships that could be construed as a potential conflict of interest.

## Publisher’s Note

All claims expressed in this article are solely those of the authors and do not necessarily represent those of their affiliated organizations, or those of the publisher, the editors and the reviewers. Any product that may be evaluated in this article, or claim that may be made by its manufacturer, is not guaranteed or endorsed by the publisher.
